# Comparison of mental health outcomes in seropositive and seronegative adolescents during the COVID19 pandemic

**DOI:** 10.1038/s41598-022-06166-y

**Published:** 2022-02-10

**Authors:** Judith Blankenburg, Magdalena K. Wekenborg, Jörg Reichert, Carolin Kirsten, Elisabeth Kahre, Luise Haag, Leonie Schumm, Paula Czyborra, Reinhard Berner, Jakob P. Armann

**Affiliations:** 1 Department of Pediatrics, University Hospital and Medical Faculty Carl Gustav Carus, Technische Universität Dresden, Fetscherstrasse 74, 01307 Dresden, Germany; 2 Biological Psychology, Institute of Psychology, Technische Universität Dresden, Zellescher Weg 19, 01069 Dresden, Germany

**Keywords:** Infectious diseases, Viral infection

## Abstract

Post-COVID19 complications such as pediatric inflammatory multisystem syndrome (PIMS) and Long-COVID19 move increasingly into focus, potentially causing more harm in young adolescents than the acute infection. To better understand the symptoms of long-term mental health outcomes in adolescents and distinguish infection-associated symptoms from pandemic-associated symptoms, we conducted a 12 question Long-COVID19 survey. Using this survey, we compared the responses on neurocognitive, general pain and mood symptoms from seropositive and seronegative adolescents in a cross-sectional study design. Since May 2020, students grade 8–12 in fourteen secondary schools in Eastern Saxony were enrolled in the SchoolCovid19 study. Serostatus was assessed regularly in all participants. In March/April 2021, 1560 students with a median age of 15 years participated at the regular study visit after re-opening of the schools in mid-March and responded to our Long-COVID19 survey as part of this visit. 1365 (88%) students were seronegative, 188 (12%) were seropositive. Each symptom asked in the Long-COVID19 survey was present in at least 35% of the students within the last seven days before the survey. With the exception of seropositive students being less sad, there was no significant difference comparing the reported symptoms between seropositive students and seronegative students. The lack of differences comparing the reported symptoms between seropositive and seronegative students suggests that Long-COVID19 might be less common than previously thought and emphasizes on the impact of pandemic-associated symptoms regarding the well-being and mental health of young adolescents.

**Clinical Trial Registration:** SchoolCoviDD19: Prospektive Erfassung der SARS-CoV-2 Seropositivität bei Schulkindern nach Ende der unterrichtsfreien Zeit aufgrund der Corona-Schutz-Verordnung (COVID-19), DRKS00022455, https://www.drks.de/drks_web/navigate.do?navigationId=trial.HTML&TRIAL_ID=DRKS00022455

## Introduction

Since the identification of the severe acute respiratory syndrome coronavirus 2 (SARS-CoV-2) as the cause of COVID-19^1^ in December 2019 and the beginning of the SARS-CoV-2 pandemic in Germany in July 2020 nearly 590.000 cases in children and adolescents have been reported by the Robert-Koch-Institute (RKI)^2^. In contrast to adults, children and adolescents usually have mild disease courses with a low rate of hospitalization^3–5^. Therefore, post-COVID19 complications such as pediatric inflammatory multisystem syndrome (PIMS) and Long-COVID19—with persisting symptoms 4 – 12 weeks and more than 12 weeks after an acute SARS-CoV-2infection^6^ move into focus, potentially causing more harm in this age group than the acute infection.

While multiple studies and registers have provided reliable data on epidemiology, clinical presentation, disease course and treatment options on PIMS^7–9^ to date no comparable data exists for Long-COVID19 in children and adolescents. A cross-sectional study from Italy^10^ in 129 children diagnosed with a SARS-CoV-2 infection found that more than 50% of participants had at least one persisting symptom 120 days after their infection; insomnia, pain, fatigue, and concentration difficulties were most commonly reported. Regularly updated reports from the Office for National Statistics (ONS) in the UK^11^ similarly provided data that symptoms in children and adolescents persisted at least 12 weeks after their SARS-CoV-2 infection.

These numbers are concerning and require attention; however, currently they merely show a temporal connection and not a causal relationship. Furthermore, a swiss study conducted among students with a median age of 11 years, could not detect any statistical difference in symptoms lasting more than 12 weeks after an acute SARS-CoV-2 infection between seropositive and seronegative individuals^12^.

In order to better understand the epidemiology and clinical manifestations of Long-COVID19 in children and adolescents and differentiate infection-associated symptoms from pandemic-associated symptoms, we conducted a 12 question Long-COVID19-survey in young adolescents. This cross-sectional survey assessed the occurrence of neurocognitive, pain or mood symptoms as described in patients suffering from Long-COVID19 in more than 1500 students participating in the SchoolCoviDD19 study in March and April 2021.

## Results

Of all 1560 participating students at the study visit in March/ April 2021 1365 (88%) were seronegative and 188 (12%) were seropositive. The Long-COVID19 survey was answered by 1504 (96.8%) of the participants. Each symptom, regardless of the expression, was present in more than one third of the students within the last seven days before the survey, most commonly happiness (98.7%) followed by tenseness (86.4%), listlessness (80.7%) and difficulties concentrating (79.3%). Myalgia/ arthralgia (35.6%) and reduced physical capacity (37.8%) were reported least commonly (see Tables 1, 2 for full results).

**Table 1:** Neurocognitive and pain results of seronegative (−) and seropositive (+) participants to the Long-COVID19-survey (*n*(%); Fisher’s exact test: *n* = 1504).

Serostatus	Not at all *n* (%)		Any *n* (%)		*p*	A little bit *n* (%)		Quite *n* (%)		Severe *n* (%)		Very severe *n* (%)	
	−	+	−	+		−	+	−	+	−	+	−	+
**Item**													
Difficulty concentrating	278 (20.9)	34 (19.1)	1049 (79.1)	144 (80.9)	0.62	710 (53.5)	98 (55.1)	230 (17.3)	23 (12.9)	88 (6.6)	19 (10.7)	21 (1.6)	4 (2.2)
Memory loss	709 (53.4)	87 (48.9)	619 (46.6)	91 (51.1)	0.26	478 (36.0)	60 (33.7)	104 (7.8)	18 (10.1)	28 (2.1)	12 (6.7)	9 (0.7)	1 (0.6)
Listlessness	251 (18.9)	39 (21.9)	1075 (81.1)	139 (78.1)	0.36	500 (37.7)	64 (36.0)	329 (24.8)	36 (20.2)	178 (13.4)	30 (16.9)	68 (5.1)	9 (5.1)
Headache	598 (45.1)	69 (38.8)	728 (54.9)	109 (61.2)	0.13	387 (29.2)	51 (28.7)	233 (17.6)	38 (21.3)	83 (6.3)	19 (10.7)	25 (1.9)	1 (0.6)
Abdominal pain	794 (59.8)	96 (53.9)	533 (40.2)	82 (46.1)	0.14	317 (23.9)	43 (24.2)	152 (11.5)	26 (14.6)	52 (3.9)	7 (3.9)	12 (0.9)	6 (3.4)
Myalgia/arthralgia	851 (64.1)	116 (65.2)	477 (35.9)	62 (34.8)	0.80	312 (23.5)	37 (20.8)	124 (9.3)	18 (10.1)	34 (2.6)	6 (3.4)	7 (0.5)	1 (0.6)
Reduced physical capacity	831 (62.6)	107 (60.1)	496 (37.4)	71 (39.9)	0.51	334 (25.2)	44 (24.7)	121 (9.1)	19 (10.7)	32 (2.4)	8 (4.5)	9 (0.7)	0 (0)

**Table 2:** Insomnia and mood results of seronegative (−) and seropositive ( +) participants to the Long-COVID19-survey (*n* (%); Fisher’s exact test: *n* = 1504).

Serostatus	Never *n* (%)		At least once *n* (%)		*p*	Once *n* (%)		Multiple times *n* (%)	
	−	+	−	+		−	+	−	+
**Item**									
Insomnia	450 (34.0)	66 (37.1)	874 (66.9)	112 (62.9)	0.45	418 (31.6)	53 (29.8)	456 (34.4)	59 (33.1)
Mood—sad	447 (33.7)	75 (42.1)	881 (66.3)	103 (57.9)	0.03	428 (32.2)	51 (28.7)	453 (34.1)	52 (29.2)
Mood—angry	422 (31.8)	59 (33.1)	905 (68.2)	119 (66.9)	0.73	506 (38.1)	73 (41.0)	399 (30.1)	46 (25.8)
Mood—happy	15 (1.1)	4 (2.2)	1313 (98.9)	174 (97.8)	0.29	88 (6.6)	8 (4.5)	1225 (92.2)	166 (93.3)
Mood—tense	181 (13.6)	23 (12.9)	1146 (86.4)	155 (87.1)	0.9072	498 (37.5)	72 (40.4)	648 (48.8)	83 (46.6)

**Figure 1:**
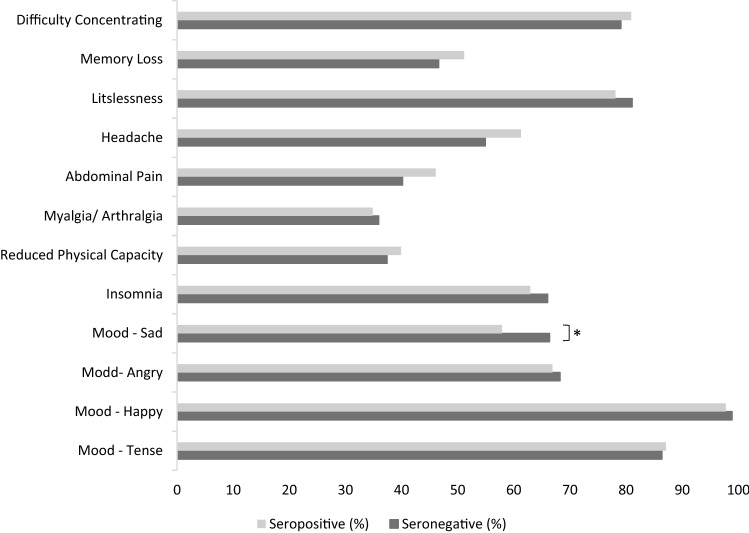
Prevalence of neurocognitive, pain and mood symptoms in seronegative and seropositive study participants (Fisher’s exact test: *n* = 1504, * *p* < .05).

Fisher’s exact test did not reveal any significant differences between seropositive and seronegative students regarding the prevalence of any of the neurocognitive and pain symptoms reported. Regarding mood symptoms, there was no differences in in happiness, tenseness or angriness, however, seropositive students were significant less likely to report sadness (Fig. 1, Table 1, 2).

To avoid underestimation of seropositive individuals due to our relatively strict definitions of seropositivity, we also analyzed the data if only the LIAISON® test result was taken into consideration for the decision on seropositivity. This resulted in 204 (13%) LIAISON®-positive and 1342 (86%) LIAISON®-negative students. There was no statistical difference (Fisher’s exact test) in the occurrence of any neurocognitive or pain symptoms between these LIAISON®-positive and -negative students either, while the lower prevalence of sadness in seropositive students could still be detected (see S*upplementals—Fig. 1*).

Spearman correlation analyses revealed that age was positively correlated with all neurocognitive, pain and mood symptoms, except for insomnia, sad mood, and angry mood. In addition, female students reported a consistently higher prevalence of neurocognitive, pain and mood symptoms compared to male students, except for Myalgia/ Arthralgia where there was no significant association with sex (Table 3).

**Table 3:** Spearman-Rho bivariate correlations between age, sex, and the reported neurocognitive, pain and mood symptoms (*n* = 1504, * *p* < .05, ** *p* < .01 (one-tailed test)).

Variable	(1)	(2)	(3)	(4)	(5)	(6)	(7)	(8)	(9)	(10)	(11)	(12)	(13)	(14)
(1) Age														
(2) Sex	−.05													
(3) Distress	.23**	.25**												
(4) Difficulty concentrating	.08**	.14**	.35**											
(5) Memory loss	.10**	.07**	.24**	.47**										
(6) Listlessness	.17**	.08**	.36**	.52**	.32**									
(7) Headache	.10**	.27**	.30**	.30**	.23**	.27**								
(8) Abdominal pain	.05*	.21**	.26**	.21**	.20**	.23**	.28**							
(9) Myalgia/arthralgia	−.07*	.05	.15**	.24**	.21**	.20**	.21**	.26**						
(10) Reduced physical capacity	.11**	.10**	.26**	.32**	.25**	.29**	.28**	.27**	.35**					
(11) Insomnia	.04	.20**	.30**	.28**	.23**	.22**	.29**	.18**	.18**	.24**				
(12) Sad	.04	.38**	.48**	.35**	.27**	.33**	.30**	.28**	.18**	.27**	.37**			
(13) Angry	−.04	.12**	.24**	.23**	.20**	.22**	.17**	.18**	.14**	.16**	.23**	.33**		
(14) Happy	−.05*	.06*	−.11*	−.17**	−.12**	−.17**	−.06*	−.05*	−.07**	−.11**	−.12**	−.11**	−.06*	
(15) Tense	.14**	.16**	.33**	.29**	.23**	.30**	.20**	.20**	.14**	.23**	.25**	.36**	.28**	−.07**

Partial correlation analyses, which were performed to test for age and sex independent effects of the analyzed serostatus on rank-transformed neurocognitive, pain and mood symptoms also revealed differences only with respect to sadness; being seronegative was associated with an increased prevalence of sadness (*n* = 1553; *p* 0.025) (Table 4).

**Table 4:** Partial correlations between serostatus and neurocognitive, pain and mood symptoms (rank-transformed), controlling for age and sex (*n* = 1504, * *p* < .05, ** *p* < .01 (one-tailed test)).

Variable	(1)	(2)	(3)	(4)	(5)	(6)	(7)	(8)	(9)	(10)	(11)	(12)	(13)
(1) Serostatus													
(2) Mental distress	−.01												
(3) Difficulty concentrating	.02	.33**											
(4) Memory loss	.05	.22**	.47**										
(5) Listlessness	−.01	.34**	.51**	.31**									
(6) Headache	.05	.24**	.27**	.21**	.24**								
(7) Abdominal pain	.05	.22**	.18**	.18**	.22**	.23**							
(8) Myalgia/arthralgia	< −.01	.16**	.23**	.21**	.20**	.20**	.26**						
(9) Reduced physical capacity	.02	.23**	.31**	.24**	.27**	.25**	.25**	.35**					
(10) Insomnia	−.02	.26**	.26**	.22**	.20**	.25**	.14**	.18**	.23**				
(11) Sad	−.06*	.44**	.32**	.25**	.32**	.22**	.22**	.17**	.25**	.32**			
(12) Angry	−.02	.22**	.22**	.20**	.21**	.15**	.18**	.13**	.15**	.22**	.31**		
(13) Happy	.01	−.12**	−.17**	−.12**	−.17**	−.07**	−.07*	−.08**	−.11**	−.13**	−.14**	−.07*	
(14) Tense	−.02	.28**	.27**	.22**	.28**	.16**	.17**	.15**	.21**	.22**	.31**	.27**	−.07**

104 out of 188 seropositive students (55%) had previously been tested positive for SARS-CoV-2 and/ or reported a confirmed SARS-CoV-2 positive household member and were therefore considered as known SARS-CoV2 infections. Compared to those with an unknown infection (84/188 (45%)) Fisher’s exact test did not reveal any significant differences regarding the prevalence of any of the neurocognitive, pain and mood symptoms reported (Tables 5, 6).

**Table 5:** Neurocognitive and pain results of seropositive participants to the Long-COVID19-survey—known vs. previously unknown infection (*n* (%); Fisher’s exact test: *n* = 188).

	None		Any		*p*
	Known infection	Unknown infection	Known infection	Unknown infection	
Difficulty concentrating	22 (22.5)	12 (15.0)	76 (77.6)	68 (85.0)	0.25
Memory loss	47 (48.0)	40 (50.0)	51 (52.0)	40 (50.0)	0.88
Listlessness	21 (21.4)	18 (22.5)	77 (78.6)	62 (77.5)	1.00
Headache	40 (40.8)	29 (36.3)	58 (59.2)	51 (63.8)	0.54
Abdominal pain	55 (56.1)	41 (51.2)	43 (43.9)	39 (48.8)	0.55
Myalgia/arthralgia	64 (65.3)	52 (65.0)	34 (34.7)	28 (35.0)	1.00
Reduced physical capacity	61 (62.2)	46 (57.5)	37 (37.8)	34 (42.5)	0.54

**Table 6:** Insomnia and mood results of seropositive participants to the Long-COVID19-survey—known vs. previously unknown infection (*n* (%); Fisher’s exact test: *n* = 188).

	Never		At least once		*p*
	Known infection	Unknown infection	Known infection	Unknown infection	
Insomnia	35 (35.7)	31 (38.8)	63 (64.3)	49 (61.3)	0.76
Mood—sad	41 (41.8)	34 (42.5)	57 (58.2)	46 (57.6)	1.00
Mood—angry	30 (30.6)	29 (36.3)	68 (69.4)	51 (63.8)	0.52
Mood—happy	1 (1.0)	3 (3.8)	97 (99.0)	77 (96.3)	0.33
Mood—tense	11 (1.22)	12 (15.0)	87 (88.8)	68 (85.0)	0.51

The median score of overall self-reported mental distress was 4 and did not differ between seropositive and seronegative participants (*n* = 1553; *p* 0.91).

## Discussion

The aim of our study was to investigate the presence of certain neurocognitive, pain and mood symptoms, being frequently reported with the occurrence of Long-COVID19 in children and adolescents in the current literature, in SARS-CoV-2 seropositive and seronegative students.

When looking at all data—regardless of the serostatus—our study clearly shows a high rate of neurocognitive, pain and mood symptoms in the surveyed group of adolescents, with every item being present in at least one third of the students within the last seven days before responding to the survey. This is consistent with previous studies and surveys on the prevalence of Long-COVID19 symptoms^10^ but also with publications regarding psychosomatic symptoms during the SARS-CoV-2 pandemic^13^ in this age group. Furthermore the overall prevalence is considerably higher compared to pre-pandemic data^13^.

When comparing the adolescents' responses according to their serostatus, we found no significant difference in eleven out of twelve of the symptoms queried. This general equal prevalence of neurocognitive, pain and mood symptoms in seronegative and seropositive adolescents in our study does not negate the existence of Long-COVID19 symptoms in general or in the pediatric population. However, it does suggest that they occur less frequently than previously assumed—at least in children and adolescents with only mild to asymptomatic courses of disease—as were investigated by this study.

Furthermore, it confirms the negative effects of lockdown measures on mental health and well-being of children and adolescents^14^. These effects—affecting this whole age group—need to be balanced with the risk of Long-COVID19 in infected individuals. This balancing act will be a difficult task for public health officials and political officials. Nevertheless, it will be a necessary one when aiming to improve mental health in adolescents.

The differentiation between infection-associated and pandemic-associated symptoms is important because the approach to mediate these symptoms will be different. While strict lock-down measures including school closures will prevent SARS-CoV-2 transmissions in this age group and thereby prevent long-term infection related illnesses, these measures will also further restrict social contact, self-determination, education and development of the affected children and adolescents and thereby amplify pandemic- or lockdown-associated symptoms.

The interpretation of the negative correlation of sadness and positive serostatus is difficult and should not be overstated given the fact that this was an exploratory study design and only self-reported symptoms are used. One could speculate that students who already had a SARS-CoV-2 infection are more understanding towards pandemic control measures and might be less fearful regarding contracting the virus in the future and therefore less sad overall. However, further studies are needed to elucidate this finding.

Neither after adjusting our results for age and sex—which are correlating with the majority of neurocognitive, pain and mood symptoms as supported by current literature^15–17^ nor after adjusting our relatively strict serostatus definition, nor when evaluating the results according to the knowledge about the SARS-CoV-2 infection, did we see different results. This supports our findings that the adolescents which participated at our study visit in March/April 2021—regardless of their serostatus—are currently very impacted by the pandemic.

While self-reported symptoms cannot be equated with the diagnosis of an illness, a prevalence of at least 35% for each symptom is a concerning screening result that requires further investigation. In addition, validated, reliable tests are needed to evaluate symptom severity in affected individuals. The fact that self-reported overall mental distress did not differ significantly between seropositive and seronegative individuals does not suggest though that infection-associated symptoms are necessarily more severe than pandemic-associated symptoms.

As a positive takeaway the fact that happiness is by far the most common response in our survey is reassuring und clearly points to the resilience of this age group.

Our study can provide a control group to SARS-CoV-2 infected adolescents by comparing the responses of seropositive individuals with those of their seronegative peers. This is crucial for the question of this study, as children and adolescents are currently exposed not only to possible long-term consequences of infection, but also to the negative effects of pandemic measures.

To capture symptoms independent of the SARS-CoV-2 infection status and thus to ensure the comparability of responses between seropositive and seronegative participants, we explicitly asked about symptoms within the past seven days before the survey.

Given the design of our SchoolCoviDD19 study with serial assessments of serostatus we can determine the progression of seropositivity among the 1560 students participating in our Long-COVID19 survey in March/ April 2021. In total, we detected 9, 14 and 188 seropositive participants in May/ June 2020, November 2020 and March/ April 2021, respectively^18,19^. The majority (*n* = 174; 92.6%) of our survey participants became infected with SARS-CoV-2 within the last 4 months before the March/April study visit. This allows us to make a rough temporal classification of the reported mental health outcomes depending on the time of infection.

There are several limitations to our study. The sample size of 188 infected individuals is not large enough to detect rare symptoms and a screening questionnaire cannot reliably compare the severity of symptoms in affected individuals. Furthermore, our questionnaire is not specifically validated for Long-COVID19 in adolescents and concentrated on neurocognitive, general pain and mood symptoms. Symptoms like a persistent sore throat, persistent cough or chest tightness and an altered sense of smell/ taste were not included.

However, to our knowledge, there are no Long-COVID19-specific questionnaires to date and furthermore Long-COVID19 symptoms are not specific. Our survey covers a variety of symptoms reported in the context of Long-COVID19 and having a control group of age-matched peers who never had a SARS-CoV-2 infection adds valuable information to the Long-COVID19 discussion that is urgently needed. In addition, even with a limited number of participants, symptoms with a prevalence of 10–30% in all infected individuals—as previously reported^10,11^ would be reliably detectable in our study.

To conclude, in our cohort of adolescents more than one third reported the presence of at least one neurocognitive, pain or mood symptom with tenseness, listlessness and difficulties concentrating being reported most commonly. However, seropositive students are not more likely to report these symptoms compared to seronegative students. Leading to the conclusion that symptoms of Long-COVID19 might be less common than previously assumed and emphasizing on the impact of pandemic-associated symptoms regarding the well-being and mental health of young adolescents.

## Methods

### Study design/demographic data

Since May 2020 students grade 8–12 in fourteen secondary schools in Eastern Saxony are enrolled in the SchoolCoviDD19 study. Two of these 14 schools are vocational schools. Seroprevalence is assessed via serial SARS-CoV-2 antibody testing in all participants. The first seroprevalence assessment was performed in May/ June 2020, followed by an assessment in October 2020 and then again in March/ April 2021. 1560 students with a median age of 15 years participated at the study visit in March/April 2021 and had their serostatus analyzed. Seven already vaccinated students were excluded from the analysis. Median age, sex and household size did not differ significantly between seropositive and seronegative participants (*n* = 1553; *p* 0.89, 0.93, 0.78) (Supplementals—Table 1).

During the March/ April 2021 study visit all participants were additionally asked to complete a survey regarding neurocognitive, pain or mood symptoms.

### Survey details

The Survey included besides sociodemographic variables (i.e., age, sex) twelve questions on the occurrence and frequency of relevant neurocognitive, pain and mood symptoms, namely difficulties concentrating, memory loss, listlessness, headache, abdominal pain, myalgia/arthralgia, reduced physical capacity, insomnia and mood (sadness, anger, happiness and tenseness) within the last seven days before the survey.

The questions were taken from the Symptom Checklist-90-R (SCL-90-R)^20^, the Somatic Symptom Scale (SSS-8)^21^ and a questionnaire about stress and stress management in children and adolescents (SS KJ 3–8 R)^22^. All questionnaires are validated in adolescents.

Answers were coded on a categorical scale—“never”, “once”, “multiple times” for insomnia and all mood questions; “not at all”, “a little bit”, “quite”, “severe” and “very severe” for the remaining questions.

In addition, a self-generated item was used to assess the overall level of mental distress on a scale from 0 (“not at all”) to 10 (“total”).

### Laboratory analysis

After informed consent was obtained from all participants and/or their legal guardian(s), a 5 ml sample of peripheral venous blood was collected during the study visit. We then assessed anti-SARS-CoV-2 IgG antibodies in all samples using a commercially available chemiluminescence immunoassay (CLIA) technology for the quantitative determination of anti-S1 and anti-S2 specific IgG antibodies to SARS-CoV-2 (Diasorin LIAISON® SARS-CoV-2 S1/S2 IgG Assay). Antibody levels > 15.0 AU/ml were considered positive and levels between 12.0 and 15.0 AU/ml were considered equivocal.

All samples with a positive or equivocal LIAISON® test result, as well as all samples from participants with a reported personal or household history of a SARS-CoV-2 infection, were re-tested with two additional serological tests. These were a chemiluminescent microparticle immunoassay (CMIA) intended for the qualitative detection of IgG antibodies to the nucleocapsid protein of SARS-CoV-2 (Abbott Diagnostics® ARCHITECT SARS-CoV-2 IgG—an index (S/C) of < 1.4 was considered negative whereas one > / = 1.4 was considered positive) and an ELISA detecting IgG against the S1 domain of the SARS-CoV-2 spike protein (Euroimmun® Anti-SARS-CoV-2 ELISA—a ratio < 0.8 was considered negative, 0.8–1.1 equivocal, >1.1 positive).

Participants whose positive or equivocal LIAISON® test result could be confirmed by a positive test result in at least one additional serological test were considered seropositive. Participants with a negative LIAISON® test result, but positive results in both additional serological tests were also considered positive.

### Statistical analysis

Results for continuous variables are presented as medians with interquartile ranges (IQR) and categorical variables as numbers with percentages, unless stated otherwise.

Fisher’s exact test was used to determine categorical variables for the statistical analysis. Thereby, the answers to the items assessing neurocognitive, pain and mood symptoms, were dummy-coded, enabling a comparison of the answer category “none” (coded 0) against the answer categories “a little bit”/“quite”/“severe”/“very severe” and “once”/“multiple times” (coded 1), respectively.

Furthermore, data distributions of the neurocognitive, pain and mood symptoms were tested for normality using the Kolmogorov–Smirnov (K-S) test. Data with distributions significantly different (*p* < 0.05) from normal were either transformed to ranks to allow parametric statistics or analyzed using non-parametric statistics.

In order to examine associations between sociodemographic variables (i.e., age, sex) and the neurocognitive, pain and mood symptoms, bivariate correlation analyses were conducted.

In a second step, partial correlation analyses were conducted between serostatus and the neurocognitive, pain and mood symptoms, adjusting for age and sex.

Analyses were performed using IBM SPSS 27.0 or Microsoft Excel 2010. All statistical tests were conducted with α < 0.05.

### Approval

The SchoolCoviDD19 study was approved by the Ethics Committee of the Technische Universität (TU) Dresden (BO-EK-156042020) and has been assigned clinical trial number DRKS00022455 (https://www.drks.de/drks_web/navigate.do?navigationId=trial.HTML&TRIAL_ID=DRKS00022455). All methods were carried out in accordance with relevant guidelines and regulations.

## Data sharing

Deidentified individual participant data will be made available, in addition to study protocols, the statistical analysis plan, and the informed consent form. The data will be made available upon publication to researchers who provide a methodologically sound proposal for use in achieving the goals of the approved proposal. Proposals should be submitted to corresponding author (jakob.armann@uniklinikum-dresden.de).

## Role of the funding source

The funder of the study had no role in the study design, data collection, data analysis, data interpretation, or writing of the report and in the decision to submit the paper for publication.

## Supplementary Information

Supplementary Information.

## Abbreviations

COVID-19: Coronavirus disease 2019
ELISA: Enzyme-linked immunosorbent assay
IgG: Immunoglobulin G
IQR: Interquartile range
PCR: Polymerase chain reaction
PIMS: Pediatric inflammatory multisystem syndrome
RKI: Robert-Koch-Institute
SARS-CoV-2: Severe acute respiratory syndrome coronavirus type 2
