# Supplementary material for: Comparison of mental health outcomes in seropositive and seronegative adolescents during the COVID19 pandemic
Source: Sci Rep. 2022 Feb 10;12:2246. doi: 10.1038/s41598-022-06166-y (PMC8831534; doi:10.1038/s41598-022-06166-y)
Supplement: Supplementary file 1 — Supplementary Information. [file 41598_2022_6166_MOESM1_ESM.pdf]

Comparison of mental health outcomes in seropositive and seronegative adolescents during the COVID19 pandemic

Judith Blankenburg[1], MD, Magdalena K. Wekenborg[2], PhD, Jörg Reichert[1], PhD, Carolin Kirsten[1], Elisabeth Kahre[1], MD, Luise Haag[1], MD, Leonie Schumm[1], MD, Paula Czyborra[1], MD, Reinhard Berner[1], MD, Jakob P. Armann[1,*], MD

Supplementals - Figure 1 Prevalence of neurocognitive, pain and mood symptoms in LIAISON®-negative and positive study participants (Fisher's exact test: n = 1553, * $p < .05$)

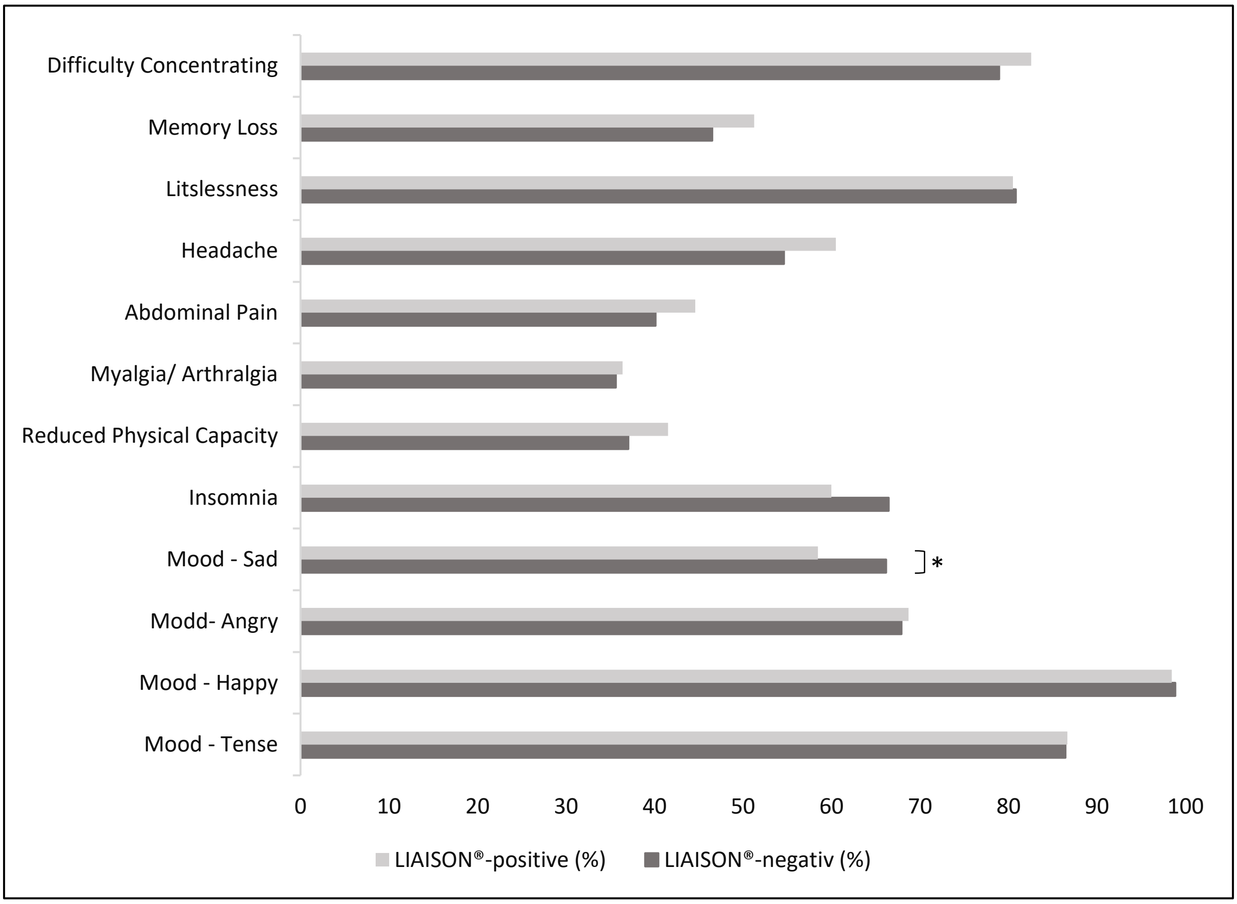

Supplemtentals - Table 1 Demographic data and serostatus of the participating students at study visit in March/ April 2021; IQR – Interquartile range (n = 1553)

	**Seronegative Students**	**Seropositive Students**
**Median Age, (IQR), (range)**	15, (14-16), (10 - 38)	15, (14-17), (10 - 35)
**Female n (%)**	762 (56)	104 (55)
**Median Household size, (IQR)**	4, (3-5)	4, (4-5)
